# Antibacterial Potential of Biosynthesized Zinc Oxide Nanoparticles against Poultry-Associated Foodborne Pathogens: An In Vitro Study

**DOI:** 10.3390/ani11072093

**Published:** 2021-07-14

**Authors:** Hidayat Mohd Yusof, Nor’Aini Abdul Rahman, Rosfarizan Mohamad, Uswatun Hasanah Zaidan, Anjas Asmara Samsudin

**Affiliations:** 1 Department of Bioprocess Technology, Faculty of Biotechnology and Biomolecular Sciences, Universiti Putra Malaysia, Serdang 43400, Selangor, Malaysia; hidayatmy@gmail.com (H.M.Y.); farizan@upm.edu.my (R.M.); 2 Bioprocessing and Biomanufacturing Research Centre, Faculty of Biotechnology and Biomolecular Sciences, Universiti Putra Malaysia, Serdang 43400, Selangor, Malaysia; 3 Department of Biochemistry, Faculty of Biotechnology and Biomolecular Sciences, Universiti Putra Malaysia, Serdang 43400, Selangor, Malaysia; uswatun@upm.edu.my; 4 Department of Animal Science, Faculty of Agriculture, Universiti Putra Malaysia, Serdang 43400, Selangor, Malaysia; anjas@upm.edu.my

**Keywords:** antibacterial, antibiotic, mechanisms, poultry, reactive oxygen species, zinc oxide nanoparticles

## Abstract

**Simple Summary:**

The overuse of antibiotics in the poultry industry has led to the emergence of multidrug-resistant microorganisms. Thus, there is a need to find an alternative to conventional antibiotics. Recently, zinc oxide nanoparticles (ZnO NPs) have gained much attention due to their excellent antibacterial activity. In addition, ZnO NPs is an essential trace mineral in poultry diets. In this sense, incorporating ZnO NPs into poultry can promote growth and performance while serving as an alternative antibacterial agent to control diseases. Therefore, this study aimed to assess the in vitro antibacterial activity and antibacterial mechanisms of ZnO NPs against poultry-associated foodborne pathogens (*Salmonella* spp., *Escherichia coli*, and *Staphylococcus aureus*). The obtained findings demonstrated effective antibacterial actions against the tested microorganisms. The nanotechnology approach could represent a new tool for combating pathogens in the poultry industry.

**Abstract:**

Since the emergence of multidrug-resistant bacteria in the poultry industry is currently a serious threat, there is an urgent need to develop a more efficient and alternative antibacterial substance. Zinc oxide nanoparticles (ZnO NPs) have exhibited antibacterial efficacy against a wide range of microorganisms. Although the in vitro antibacterial activity of ZnO NPs has been studied, little is known about the antibacterial mechanisms of ZnO NPs against poultry-associated foodborne pathogens. In the present study, ZnO NPs were successfully synthesized using *Lactobacillus plantarum* TA4, characterized, and their antibacterial potential against common avian pathogens (*Salmonella* spp., *Escherichia coli*, and *Staphylococcus aureus*) was investigated. Confirmation of ZnO NPs by UV-Visual spectroscopy showed an absorption band center at 360 nm. Morphologically, the synthesized ZnO NPs were oval with an average particle size of 29.7 nm. Based on the dissolution study of Zn^2+^, ZnO NPs released more ions than their bulk counterparts. Results from the agar well diffusion assay indicated that ZnO NPs effectively inhibited the growth of the three poultry-associated foodborne pathogens. The minimum inhibitory concentration (MIC) and minimum bactericidal concentration (MBC) were assessed using various concentrations of ZnO NPs, which resulted in excellent antibacterial activity as compared to their bulkier counterparts. *S. aureus* was more susceptible to ZnO NPs compared to the other tested bacteria. Furthermore, the ZnO NPs demonstrated substantial biofilm inhibition and eradication. The formation of reactive oxygen species (ROS) and cellular material leakage was quantified to determine the underlying antibacterial mechanisms, whereas a scanning electron microscope (SEM) was used to examine the morphological changes of tested bacteria treated with ZnO NPs. The findings suggested that ROS-induced oxidative stress caused membrane damage and bacterial cell death. Overall, the results demonstrated that ZnO NPs could be developed as an alternative antibiotic in poultry production and revealed new possibilities in combating pathogenic microorganisms.

## 1. Introduction

The unrestricted overuse of antibiotics promotes multidrug resistance in microorganisms, compromising human and animal health. Hence, there is a pressing need to develop alternatives to traditional antimicrobials that are more effective with new action mechanisms. One of the current alternatives to combat multidrug-resistant pathogens is nanobiotics, also known as nanoparticles (NPs) with antimicrobial properties. Nanoparticles currently gained much attention due to their unique characteristics, which offer many explicit properties for biomedical applications [1]. Inorganic NPs, such as gold (AuNPs), silver (AgNPs), zinc oxide (ZnO NPs), and selenium NPs (SeNPs), have profound applications in medical and biological fields such as medical diagnosis, biosensor, and personal care products and have been widely explored as antibacterial agents [2,3,4] due to their distinctive physicochemical and biological properties over their bulk phase.

Among the NPs, ZnO NPs are specifically vital as efficient metal oxide NPs and exhibited various antibacterial properties against a wide range of microorganisms, including Gram-positive and Gram-negative bacteria, as well as major foodborne pathogens. The properties of ZnO NPs, such as extensive surface area, biocompatibility, biodegradability, semiconductor behavior, and UV light barrier, render their vast application. In addition, ZnO NPs have been utilized as antimicrobial agents in food packaging [5], topical creams, and antibiotic agents in animal feed due to their strong bactericidal effect associated with their small particles and higher surface energies [1].

Zinc (Zn) is an important trace element for poultry required for the growth, health, and metabolic function of the body [6]. However, the Zn content in the raw material diet in poultry feed is too low to meet the poultry requirements. Thus, Zn supplementation in the form of inorganic Zn has been widely used. However, the major drawbacks of using inorganic Zn is its poor bioavailability and utilization rate [7], causing the feed manufacturers to apply a greater amount of dietary Zn (100 to 120 mg/kg feed) to achieve the maximum performance of poultry, which is above the recommended standard (40 mg/kg) by the National Research Council (NRC, 1994) [8]. Nonetheless, the extensive use of high-dose dietary zinc oxide (ZnO) may affect the stability of other trace elements [9,10] and lead to excess Zn in excreta, causing environmental contamination. With the emergence of nanotechnology, which is associated with the smallest particle size ranging from 1 to 100 nm, the bioavailability and absorption efficiency of Zn were improved. Therefore, the effects of ZnO NPs have been prominently studied as a feed supplement in the poultry industry to improve the bioavailability of Zn in the body [9,10,11] and reduce the excretion of Zn in feces. Aside from promoting poultry growth and performance, the supplementation of ZnO NPs also received great attention for its antibacterial effects. Furthermore, many studies have shown the effectiveness of ZnO NPs in controlling the gut microbiota in poultry and livestock [12,13,14,15], demonstrating its potential use as an antibiotic. In this sense, introducing ZnO NPs into poultry feed could potentially serve as an antibiotic, as well as a Zn supply.

Several challenges emerged with the global exponential growth of broiler meat consumption, including infectious diseases caused by poor biosecurity and husbandry practices [16]. Microbial diseases caused by pathogens mainly contribute to the high mortality in the poultry industry. In poultry production, different kinds of antibiotics are used to control diseases and promote body growth performance. However, frequent use of antibiotics in the poultry industry contributed to major health threats in the society associated with antibiotic residue in meat, eggs, and other animal products [16]. *Salmonella*, *Escherichia coli*, and *Staphylococcus aureus* are major poultry product contaminants associated with human foodborne pathogen illness [17,18] and a major cause of opportunistic human and animal infections. These pathogenic bacteria commonly colonize poultry and could also lead to serious consequences if not treated. *Salmonella*, for example, is one of the most prevalent foodborne pathogens that can spread from contaminated poultry products such as meat and eggs to humans, resulting in salmonellosis [19]. Therefore, lowering the burden of poultry-associated foodborne pathogens in the gut of poultry could help reduce the contamination of poultry products as well as diseases caused by these pathogens. As more pathogenic bacteria develop resistance to conventional antibiotics, nanotechnology offers new paths for antibacterial drug design.

Scientific evidence demonstrated the potential of ZnO NPs as an alternative to conventional antibiotics in livestock farming. Yausheva et al. [12] conducted an intestinal microbiome assessment on the supplementation of ZnO NPs in broiler chicken. The results show that the ZnO NPs supplementation resulted in a decreased number of pathogenic microorganisms in broiler chicken cecum. Likewise, Wang et al. [20] reported that ZnO NPs supplementation in piglets diet could reduce diarrhea caused by some common bacterial such as *E. coli* and *Salmonella* than high dietary ZnO. The study suggested that ZnO NPs could be used as a feed antibiotic and to replace high dietary ZnO levels. However, the exact antimicrobial mechanisms of ZnO NPs are not completely understood, and the most common antibacterial action of ZnO NPs is attributed through multifaceted mechanisms, including the generation of ions from the surface of NPs [1]. The ions will bind to electron donor groups on the bacterial cell surface and subsequently damage the bacterial cell membrane [21]. Besides, the intrinsic physicochemical properties of ZnO NPs allow for the generation of reactive oxygen species (ROS), which induces oxidative stress and cell death [22].

The biological synthesis of metal nanoparticles (NPs) has many advantages compared to physical and chemical synthesis due to their eco-friendliness, biocompatible properties, and low cost. Although chemical approaches outperform biological alternatives in terms of production rate and NPs size control, chemically synthesized NPs are less biocompatible due to the usage of toxic chemicals for capping and stabilizing agents. Triethylamine, thioglycerol, and ethylenediaminetetraacetic acid (EDTA) [23] are commonly employed as agents in the capping and stabilizing process to control the size of NPs and prevent their aggregation. Nonetheless, these chemicals may reside or bind with the final product of NPs, and their presence is considered to have an adverse impact when used in biological applications. This issue does not arise when NPs are synthesized using biological synthesis routes such as by plant extract or microorganisms. Biological synthesis does not require external chemical sources for reducing and stabilizing, as the reactions are mediated by the biological molecules present within the system [6,24]. Moreover, the production of ZnO NPs using biological molecules is of interest for sustainable production and biomedical applications, in line with the green chemistry prospect. A previous study by Darvishi et al. [25] compared the cytotoxicity effect between biosynthesized (walnut extract) and chemically synthesized ZnO NPs on human skin fibroblasts and found that the biosynthesized ZnO NPs were less toxic to the cells. They discovered that the hazardous capping agent in chemically synthesized ZnO NPs causes cytotoxicity, while biomolecules from walnut extract serve as a capping agent, reducing toxicity.

The present work involves the biological synthesis of ZnO NPs using *Lactobacillus plantarum* TA4 from our previous study [26]. The biosynthesized ZnO NPs were analyzed by UV-Visible spectroscopy, and the size and shape morphology was determined using a high-resolution transmission electron microscope (HR-TEM). To date, studies related to the efficacy of ZnO NPs against poultry-associated foodborne pathogens are scarce. Hence, the present study attempted to assess the in vitro antibacterial potential of ZnO NPs against poultry-associated foodborne pathogen isolates of *Salmonella*, *E. coli*, and *S. aureus* for their potential application in combating pathogens in the poultry industry. The in vitro antibacterial potential of ZnO NPs was investigated through the determination of the inhibition zone using the agar well diffusion method. The minimum inhibitory concentration (MIC) and minimum bactericidal concentration (MBC) and time-killing assay of ZnO NPs were also determined. Trypan blue exclusion assay, quantification of protein, reducing sugar, ROS formation, and cell morphological changes using a scanning electron microscope (SEM) were performed to investigate the antibacterial mechanisms of ZnO NPs.

## 2. Materials and Methods

### 2.1. Bacterial Strain and Media

Three poultry-associated foodborne pathogenic isolates, i.e., *Salmonella* spp., *E. coli*, and *S. aureus* isolated from the small intestine of broiler chicken, were obtained from the Department of Animal Science, Universiti Putra Malaysia. The isolates were grown overnight in nutrient broth at 37 °C. A previously isolated zinc-tolerant *L. plantarum* TA4 was used for the biosynthesis of ZnO NPs [27]. The strain was grown in the de Man, Rogosa and Sharpe (MRS) (Oxoid™, Basingstoke, UK) broth and incubated for 24 h at 37 °C on an orbital shaker at 150 rpm. After the incubation, the supernatant was collected by centrifugation at 10,000× *g* for 5 min.

### 2.2. Zinc Oxide Nanoparticles Preparation and Characterization

The established procedure from our previous study [26] was followed for the biosynthesis of ZnO NPs with some modifications. Briefly, 20 mL of supernatant from zinc-tolerant *L. plantarum* TA4 was mixed with 80 mL of 500 mM aqueous zinc nitrate (Zn(NO_3_)_2_•6H_2_O) solution. The mixture was continuously stirred with a magnetic stirrer for 4 h at room temperature. The formation of ZnO NPs was confirmed by the appearance of white coalescence in the reaction mixture. Subsequently, the biosynthesized ZnO NPs were separated using centrifugation (10,000× *g* for 20 min) and washed with dH_2_O and ethanol multiple times, and drying overnight at 100 °C to obtain the white powder of ZnO NPs.

ZnO NPs were characterized by physicochemical methods. The surface plasmon resonance (SPR) was characterized by UV-Visual spectroscopy (Uviline 9400, Secomam, Alès, France) in the range of 300 to 600 nm and 1 nm resolution. The morphology of ZnO NPs was observed by HR-TEM (JEM-2100F, JEOL, Tokyo, Japan). The average particle size of ZnO NPs was determined by measuring at least 300 particles using ImageJ software (National Institute of Health, Bethesda, MD, USA).

### 2.3. Determination of Zn^2+^ Dissolution

The concentrations of dissolved Zn^2+^ from ZnO NPs were measured following the method described by Haque et al. [28]. A 100 µg/mL of ZnO NPs or bulk ZnO was suspended in 0.85% NaCl. The suspensions were incubated at room temperature for 24 h on an orbital shaker at 150 rpm. About 1 mL of sample was withdrawn at intervals of 0, 2, 4, 6, 8, 10, 12, and 24 h and centrifuged (20,000× *g* for 10 min). The zinc concentration in the media was determined by inductively coupled plasma atomic emission spectroscopy (ICP-OES) (Optima 3700, Perkin Elmer, Waltham, MA, USA).

### 2.4. In Vitro Antibacterial Activity of Biosynthesized Zinc Oxide Nanoparticles

#### 2.4.1. Agar Well Diffusion Method

Agar well diffusion method was used to investigate the antibacterial activity of biosynthesized ZnO NPs against poultry-associated foodborne pathogens following the procedure outlined in our previous study [26]. Briefly, the bacterial strains were grown until they reached the 0.5 McFarland turbidity standards and a lawn of bacterial strain was made by spreading them uniformly onto a nutrient agar (NA) (Merck, Darmstadt, Germany) plate using a sterile cotton swab. A sterile cork borer of 6 mm in diameter was used to make the wells, and about 100 µL of ZnO NPs (at concentrations of 1000, 2000, 3000, 4000, and 5000 µg/mL) were filled into respective wells. The agar plates were incubated for 24 h at 37 °C, and the diameter (mm) of inhibitory activity shown by a clear zone around each well was measured with a ruler. Bulk ZnO served as a negative control. The experiments were carried out in triplicate.

#### 2.4.2. Minimum Inhibitory Concentration (MIC) and Minimum Bactericidal Concentration (MBC) of Biosynthesized Zinc Oxide Nanoparticles

The MIC is the lowest concentration of a particular antibacterial agent that could inhibit bacterial growth. The MIC of biosynthesized ZnO NPs against the bacteria was examined using 2,3,5-triphenyl tetrazolium chloride (TTC) (Merck, Darmstadt, Germany) in a 96-well microtiter plate, according to the Clinical and Laboratory Standards Institute (CLSI) guidelines [29] with some modifications as a previously described method by Ashengroph et al. [30]. Briefly, the bacterial culture was grown until they reach a 0.5 McFarland standard. Then, 10 µL of bacterial suspension was pipetted to the wells containing 140 µL of nutrient broth containing various concentrations of ZnO NPs (10 to 5000 µg/mL). Nutrient broth without ZnO NPs was served as a control. The microtiter plate was incubated for 24 h at 37 °C. Subsequently, about 10 µL of TTC solution with an initial concentration of 20 mg/mL was added to each well and then incubated for 3 h at 37 °C. The MIC value was considered in the wells without the red color formation.

Meanwhile, the MBC is the lowest concentration of antibacterial agent that fully kills the bacteria, where no bacterial growth is observed. The MBC was assessed by sub-culturing the well suspension from MIC results onto nutrient agar aseptically. Briefly, about 10 µL of bacterial suspension was dropped on the agar plate and incubate for 24 h at 37 °C. The lowest concentration that did not display any bacterial growth was considered as the MBC. All experiments were carried out in triplicate.

#### 2.4.3. Antibiofilm Activities of Zinc Oxide Nanoparticles

##### Biofilm Inhibition Assay

The potential of ZnO NPs to inhibit initial cell attachment was determined using a biofilm inhibition assay following the method described by Famuyide et al. [31]. Briefly, the inhibitory activity was measured using a 96-well microtiter plate filled with 180 µL nutrient broth (Merck, Darmstadt, Germany). A 10 µL inoculum, with the OD_560_ = 1.0 of *Salmonella* spp., *E. coli*, and *S. aureus* was pipetted to the individual broth and incubated for 6 h at 37 °C without shaking. After incubation, a series of ZnO NPs concentration (final concentration of 0.5×, 2×, 4×, and 8× MIC) were added into the wells and further incubated for 24 h at 37 °C in a static condition. After incubation, the cultures were gently discarded and rinsed three times with PBS to remove free-floating cells before air-dried in the laminar flow. The wells were stained with 100 µL 0.1% (*w*/*v*) crystal violet and after the incubation at room temperature for 15 min, the dye was discarded and washed with dH_2_O repeatedly and dried at 60 °C for 30 min. Finally, the dye was destained with 95% ethanol and allowed to sit for 30 min, and the OD of the biofilm formed associated with crystal violet was determined at 590 nm. The untreated wells served as the control. The assay was conducted in triplicate, and the percentage of biofilm inhibition was calculated using the following formula:

$$Biofilm\;inhibition\;\left(\%\right)=\frac{\left(OD\;control-OD\;treatment\right)}{OD\;control}\times 100$$

##### Biofilm Eradication Assay

Similar to the biofilm inhibition assay, the bacterial cells were added into each well of a 96-well microtiter plate and incubated at 37 °C for 24 h (irreversible attachment phase) and 48 h (mature biofilm). The plates were then incubated in a static condition to enable the formation of a multilayer biofilm. After biofilm formation for the respective incubation periods, ZnO NPs were added in the wells at concentrations corresponding to 0.5×, 2×, 4×, and 8× MIC values and were further incubated for 24 h. After incubation, the wells were washed with distilled water and stained with crystal violet according to the previously described procedure. The untreated wells served as the control. The assay was performed in triplicate, and the biofilm eradication was calculated using the following formula:

$$Biofilm\;eradiction\;\left(\%\right)=\frac{\left(OD\;control-OD\;treatment\right)}{OD\;control}\times 100$$

#### 2.4.4. Time–Kill Assay of Zinc Oxide Nanoparticles

Time–kill assay was carried out following the procedure described by Mohamed et al. [32] with modification. A time-killing assessment was conducted to evaluate the bactericidal activity of ZnO NPs on poultry-associated foodborne pathogens for 24 h. An inoculum size of 3 × 10^9^ CFU/mL of each bacterial cell was used for the time-killing assay treated with ZnO NPs. The time–kill test consisted of untreated bacteria as the control (without ZnO NPs addition) and bacteria-treated samples at series concentrations of ZnO NPs (final concentrations of 2×, 4×, and 8× MIC). The bacterial suspension was then incubated at 37 °C on an orbital shaker at 150 rpm. Aliquots of 100 µL of bacterial suspension from each treatment group were removed at time intervals of 0, 4, 8, 12, and 24 h, and plated on nutrient agar after a 100-fold dilution in 0.85% NaCl for the determination of CFU/mL by the plate count technique. The agar plates were then incubated for 24 h at 37 °C prior to colony counting. The time–kill curve was plotted as log_10_ CFU/mL against time. The assay was conducted in triplicate.

### 2.5. Assessment of Cell Membrane Integrity (Trypan Blue Exclusion Assay)

The membrane integrity of bacterial cells was determined by trypan blue exclusion assay, according to Hossain et al. [33]. Briefly, the bacterial strains were exposed to ZnO NPs at the concentration of 8× MIC following the time-killing assay procedure described earlier. About 100 µL of bacterial suspension was removed at 0 and 24 h and then mixed with 0.4% trypan blue solution at 1:1 ratio, mixed gently, and incubated for 10 min. After incubation, 20 µL of trypan blue-bacterial suspension was loaded on a microscope glass slide and air-dried, followed by viewing the live and dead cells using a phase-contrast microscope (Olympus CX21, Tokyo, Japan) at 100× magnification.

### 2.6. Quantification of Reactive Oxygen Species (ROS)

All the bacterial strains were exposed to ZnO NPs (final concentration of 2×, 4×, and 8× MIC) to evaluate the difference in intracellular ROS generation at different time intervals (0, 8, and 24 h). ROS quantification was carried out following the method previously outlined by Tiwari et al. [34] with some modifications. The bacterial pellet exposed to ZnO NPs was obtained by centrifugation at 10,000× *g* for 10 min at 4 °C. About 500 µL of 2% nitro blue tetrazolium (NBT) (Merck, Darmstadt, Germany) solution was introduced to the bacterial cell, vortexed, and incubated for 1 h in darkness. Afterward, the mixture was centrifuged (10,000× *g* for 10 min) to remove the supernatant, followed by multiple washed with PBS and centrifuged. The pellet was then suspended in 2 M KOH for cell membrane disruption, followed by the addition of 50% dimethyl sulfoxide (DMSO) solution and incubation at room temperature for 10 min to dissolve the formazan crystal. A blue-colored mixture was observed, indicating the reduction of NBT by ROS. The mixture was centrifuged and the absorbance of the supernatant was measured at 620 nm.

### 2.7. Assay for the Membrane Leakage of Protein and Reducing Sugar

The effect of ZnO NPs on membrane leakage was determined by quantifying protein and reducing sugars from the intracellular cytosol of the cells after treated with ZnO NPs. As described earlier, each bacterial strain was treated with ZnO NPs at different concentrations. The bacterial suspension was withdrawn at time intervals (0, 8, and 24 h), and the supernatant was collected by centrifugation (10,000× *g* for 5 min). The obtained supernatant was stored at −20 °C until further use. This supernatant was used to quantify protein and reducing sugars. Protein was quantified using the Bradford assay method [35] while reducing sugars was determined using the dinitrosalicylic acid assay [36].

### 2.8. Morphological Analysis of Bacterial Cell by Scanning Electron Microscope (SEM)

A SEM analysis was carried out to investigate the effects of ZnO NPs on bacterial cell morphology. Briefly, each of the bacterial cells was treated with ZnO NPs at the concentration of 8× MIC and incubated for 24 h at 37 °C on an orbital shaker at 150 rpm. The untreated cells were used as a control. Bacteria cells were collected by centrifugation and processed before viewing under SEM (JSM-IT100, JEOL, Tokyo, Japan).

### 2.9. Data Analysis

All the antibacterial activity experiments were conducted in triplicates. Data obtained were analyzed by one-way analysis of variance (ANOVA) and mean comparisons were carried out using Tukey’s test with *p* < 0.05 indicating significance. Data analysis was performed using GraphPad Prism software (Version 7.0).

## 3. Results and Discussion

### 3.1. Biosynthesized Zinc Oxide Nanoparticles Characterization

**Figure 1:**
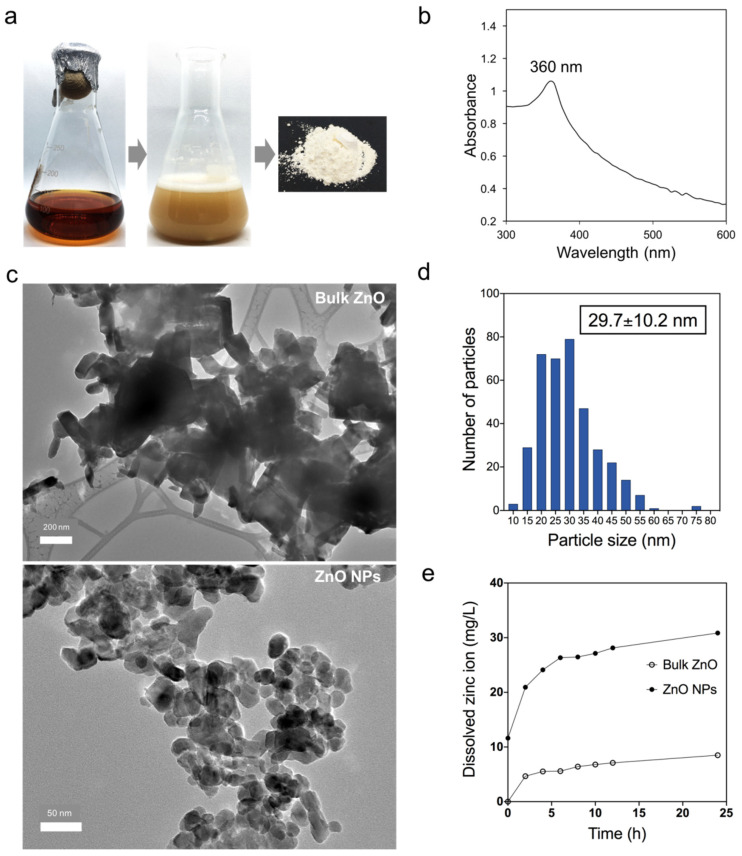
(**a**) Reduction of Zn^2+^ to ZnO NPs using *L. plantarum* TA4. (**b**) UV-Visual spectroscopy of biosynthesized ZnO NPs. (**c**) HR-TEM micrographs of bulk ZnO and biosynthesized ZnO NPs. (**d**) The size distribution of ZnO NPs (based on HR-TEM image). (**e**) Dissolution of Zn^2+^ from bulk ZnO and ZnO NPs in 0.85% NaCl suspension over incubation time assessed by ICP-OES. The concentration of Zn used in this study was 100 mg/L.

Biosynthesis of Zinc oxide nanoparticles (ZnO NPs) by using microorganisms has been vastly employed due to their eco-friendly and cost-effective method [6] that offers a highly sustainable economic alternative to the conventional synthesis method. In this present study, the biosynthesized ZnO NPs were prepared using a supernatant of *L. plantarum* TA4. We previously reported that strain TA4 could reduce Zn^2+^ to ZnO NPs both intra- and extracellularly. A whitish precipitate was observed in the CFS upon reacting with Zn^2+^, indicating the metal ion reduction and formation of ZnO NPs. The resulted ZnO NPs were collected and dried to obtain a white powder form of ZnO NPs (Figure 1a). The formation of ZnO NPs was validated using UV-Vis spectroscopy which exhibits a characteristic surface plasmon band (SPR) center at 360 nm (Figure 1b). The obtained SPR peak was in the range of the typical ZnO NPs band, as reported in the literature [37]. Furthermore, the size and shape morphology of biosynthesized ZnO NPs were characterized by HR-TEM. Figure 1c shows the HR-TEM image of bulk ZnO and ZnO NPs. The ZnO NPs displayed an oval shape with various sizes, while bulk ZnO was presented as hexagonal shaped and large aggregated particles. The average particle size of ZnO NPs was 29.7 nm as determined by ImageJ software (Figure 1d).

We hypothesized that Zn^2+^ is one of the key mechanisms for antibacterial activity; therefore, we studied the dissolved Zn^2+^ released from ZnO NPs and bulk ZnO suspended in media similar to the antibacterial assay. As shown in Figure 1e, a significant amount of Zn^2+^ was released from ZnO NPs and bulk ZnO. Both ZnO NPs and bulk ZnO were observed to continuously release ions into the media suspension, and the number of ions released increased in parallel with the incubation time. In addition, the fractions of dissolved Zn^2+^ released from ZnO NPs were higher than those released from bulk ZnO. The finding indicated that a smaller size of ZnO NPs could accelerate the release of Zn^2+^. Accumulated evidence has revealed that size has a significant impact on the rates of reactions. For instance, particles of smaller size have a larger surface area and demonstrate higher chemical reactivity; resulting in increased antibacterial efficacy as compared to their bulkier counterparts [38,39,40].

In addition, due to their small particle size, ZnO NPs have been widely used as a feed supplement in livestock and poultry industries, based on their capacity to improve the bioavailability and utilization rate of nutrients in animal bodies [6,41]. ZnO NPs also exhibited potential antibacterial activity for the control of bacterial diseases. The larger surface area of the NPs allows for more exposure to the bacterial cell surface, leading to better antibacterial activity than bulk ZnO. Although ZnO NPs are effective against a wide range of microorganisms [42], there is data paucity on their antibacterial effect on specific pathogens that are common in the livestock and poultry industry.

### 3.2. Agar Well Diffusion Assay

**Figure 2:**
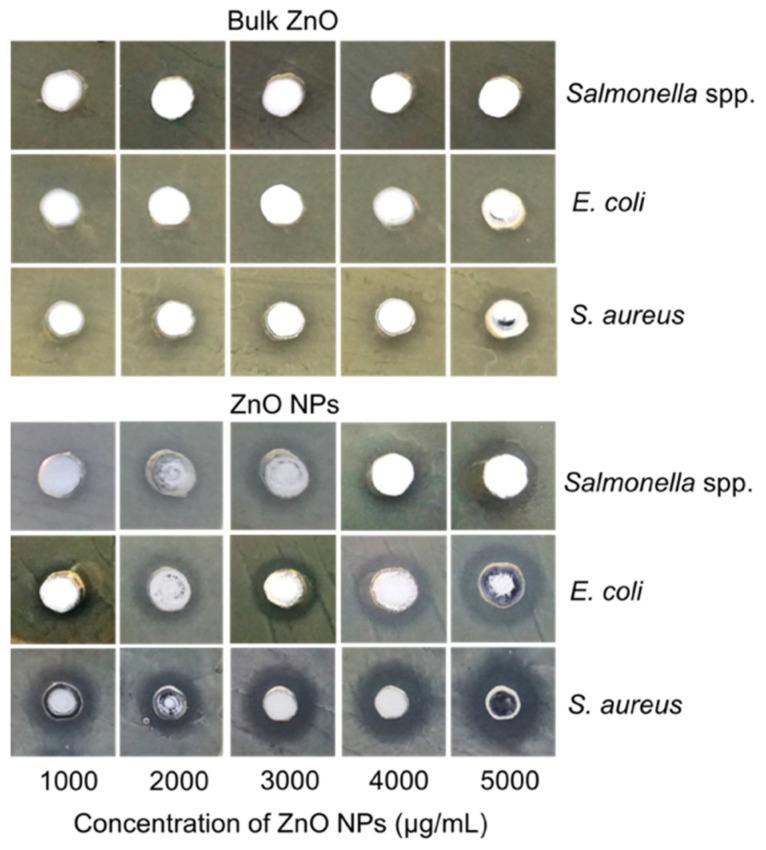
Antibacterial activity of zinc oxide nanoparticles (ZnO NPs) was investigated using the agar well diffusion method. Plates were incubated at 37 °C for 24 h, and images were taken at 24 h. Images are representative of three biological replicates.

The antibacterial property of biosynthesized ZnO NPs was examined by agar well diffusion method against three types of poultry-associated foodborne pathogens such as *Salmonella* spp., *E. coli*, and *S. aureus*, isolated from broiler chicken. These three pathogens were chosen due to their prevalence in the gastrointestinal tract of poultry. In this study, a clear bacterial growth zone was observed in wells containing ZnO NPs, with different zones of inhibition diameters at different levels of concentration against the three pathogens. The inhibitory zones are presented in Table 1. A notable increase in diameter is observed with the increment of the ZnO NPs concentration. The study revealed that the inhibitory activity value of the ZnO NPs is higher against *S. aureus* than the other two pathogens. Whereas the inhibition zones of *Salmonella* spp. and *E. coli* are more or less similar to each other (Figure 2). Bulk ZnO was used as a control to compare with the nano-sized ZnO antimicrobial activity. Notably, the well-containing bulk ZnO does not show any inhibitory activity against *Salmonella* spp. in contrast to ZnO NPs, which exhibit an increase in the inhibitory zone at concentrations of 4000 and 5000 µg/mL (*p* < 0.05). However, although no inhibition zone is observed for *E. coli* at lower ZnO concentration, inhibition zones are recorded at 4000 and 5000 µg/mL concentrations, which is significantly lower (*p* > 0.05) compared to ZnO NPs. Meanwhile, *S. aureus* is more susceptible to bulk ZnO but at a lower degree of activity than the ZnO NPs, where the inhibitory zone increases significantly (*p* < 0.05) with increasing concentration for each treatment group (Table 1).

The agar well diffusion method is commonly used to evaluate in vitro antimicrobial activity. Nonetheless, this method could not ascertain the exact mechanisms of the antibacterial capacity of ZnO NPs, as there was no direct interaction between the ZnO NPs and the bacterial cells. The inhibitory mechanisms are likely due to the free Zn^2+^ from ZnO NPs that diffuses through the agar and inhibits bacterial growth, causing the clear zone. It should be observed that Zn^2+^ is toxic to bacterial cells at higher concentrations. Furthermore, the agar well diffusion assay results corroborated with the results of the Zn^2+^ dissolution analysis (Figure 1e), which showed that Zn^2+^ release from ZnO NPs was higher than that of their bulkier counterparts, exerting their antibacterial potency.

### 3.3. Determination of Minimum Inhibitory Concentration (MIC) and Minimum Bactericidal Concentration (MBC) Value

**Figure 3:**
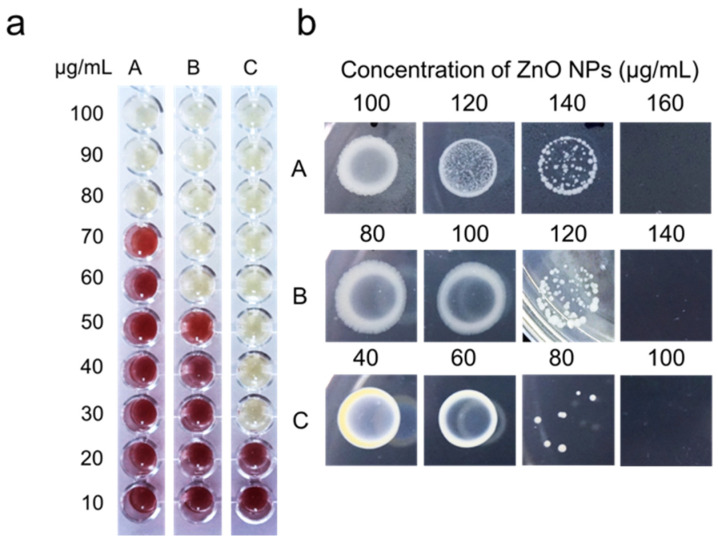
(**a**) Minimum inhibitory concentration (MIC) and (**b**) minimum bactericidal concentration (MBC) of biosynthesized ZnO NPs against (**A**) *Salmonella* spp., (**B**) *E. coli*, and (**C**) *S. aureus*.

The MIC assay was performed to determine the lowest concentration of ZnO NPs that inhibited bacterial growth, while MBC was conducted to determine the lowest concentration of ZnO NPs that killed 99.9% of the bacterial cells. Bacterial growth in the MIC assay was studied by visually inspecting the color changes from yellow to red following the addition of TTC in the culture. The changes to red formazan are directly proportional to the viable active bacterial cells. The MIC and MBC values are presented in Table 2. The MIC values of ZnO NPs against *Salmonella* spp., *E. coli*, and *S. aureus* are 80, 60, and 30 µg/mL, with the corresponding MBC values of 160, 140, and 100 µg/mL. The MIC results in Figure 3a illustrate that *S. aureus* is more vulnerable to ZnO NPs than the other tested bacteria, with a low concentration value for inhibiting bacterial growth. Meanwhile, the bulk ZnO exhibits high MIC and MBC values against the test organisms (Table 2). It should be observed that lower MIC and MBC values indicate greater antibacterial effectiveness.

As illustrated in Figure 3b, the reduction of the viable cells on the agar plate is observed with the increment of the ZnO NPs concentration within 24 h of incubation, indicating their bactericidal efficacy at a certain level of concentration, normally higher than the MIC value. Similar to the agar well diffusion results (Figure 2), the growth of *S. aureus* is efficiently repressed at lower ZnO NPs concentrations compared to *Salmonella* spp. and *E. coli*. Our findings are consistent with other studies, suggesting that Gram-positive bacteria (*S. aureus*) are more vulnerable to NPs [43,44]. The efficiency of ZnO NPs as an antibacterial agent can be associated with the cell wall properties of the microorganisms. The cell wall of Gram-positive bacteria is composed of almost 60% teichoic acids [45], an anionic glycopolymer that served as an anion site for the metal cation from ZnO NPs, elucidating why *S. aureus* is more susceptible to ZnO NPs. On the other hand, Gram-negative bacteria have two cell membranes, the plasma membrane, and the outer membrane, whereas Gram-positive bacteria only have one. The primary function of the outer membrane is to act as a selective permeability barrier, shielding the cells from any harmful compounds [4]. In addition, *Salmonella* spp. and *E. coli* are capable of producing extracellular polymeric substances (EPS) [46] that protect them from adverse environmental conditions, contributing to their resistance to a certain concentration of ZnO NPs. Nonetheless, bulk ZnO had higher MIC and MBC values, suggesting their less effective antibacterial activity against the tested bacteria.

As described earlier, the antibacterial effects of ZnO NPs are mostly attributed to Zn^2+^ [6,38]. The results demonstrated that the smaller particle size of ZnO NPs enhances the antibacterial activity due to their greater surface area to volume ratio, which enhances their surface reactivity and the release of more ions [40]. Padmavathy and Vijayaraghavan [40] demonstrated that the bactericidal efficacies of ZnO NPs with 12 nm particle size are more effective than those with a larger size, which increased the surface reactivity of ZnO NPs. In their study, the specific surface area of ZnO NPs with a size of 12 nm yielded from the Brunauer–Emmett–Teller (BET) measurement analysis was 112 m^2^g^−1^, higher than the bulk ZnO (5.11 m^2^g^−1^). They suggested that ZnO NPs with a large surface area produced more oxygen species and ions on their surface, resulting in increased antibacterial activity [40]. On the contrary, the bulk ZnO showed less bactericidal activity due to their large particles, with a small surface area; thus, releasing fewer oxygen species and ions. This observation was consistent with the data obtained from the Zn^2+^ dissolution study (Figure 1e), i.e., more Zn^2+^ was dissolved from ZnO NPs than from bulk ZnO.

### 3.4. Antibiofilm Activity of Zinc Oxide Nanoparticles

Biofilm formation is an important factor employed by bacterial pathogens to invade host cells and enhance an on-going infectious process. Biofilm is defined as bacterial groups that clump together and firmly adhere to a surface surrounded by the extracellular polymeric substance (EPS) produced by the bacteria [47]. The primary function of biofilm is to protect the microorganisms from unfavorable environments, including antibacterial agents, thus resulting in resistance to antimicrobial agents and host defenses. A serious health problem might ensue when there is a failure to either prevent or eradicate microbial biofilm. In this study, the ZnO NPs ability to inhibit and eradicate the biofilm was investigated, and the results were evaluated by the formation of a thin layer of biofilms after staining with crystal violet.

**Figure 4:**
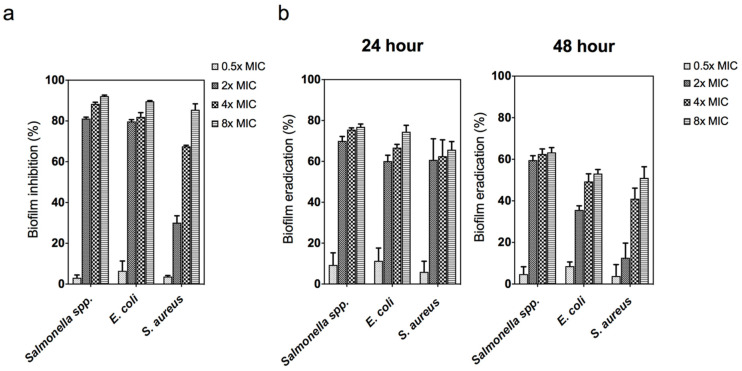
(**a**) Biofilm inhibitory following treatment with ZnO NPs 0.5×, 2×, 4×, and 8× minimum inhibitory concentration (MIC) against *Salmonella* spp., *E. coli*, and *S. aureus*. (**b**) Biofilm eradication at 24 h and 48 h following treatment with ZnO NPs 0.5×, 2×, 4×, and 8× MIC against *Salmonella* spp., *E. coli*, and *S. aureus*. Biofilm was assessed by crystal violet staining and measured at an absorbance of 590 nm. The data represent the mean ± SD of three replicates.

As presented in Figure 4a, the ZnO NPs effectively inhibited biofilm formation in all the bacteria in a dose-dependent manner. The amount of biofilm formation decreased with the increasing concentration of ZnO NPs. Moreover, lower ZnO NPs concentrations resulted in a lower percentage of biofilm inhibition (below the MIC value for each pathogen). The antibiofilm result in this study revealed that ZnO NPs could reduce biofilm formation by affecting the growth of planktonic bacteria. These results are consistent with that of Bhattacharyya et al. [48], who found that ZnO NPs reduced *Streptococcus pneumonia* cell adhesion to the surface at sub-MIC doses. Likewise, Khan et al. [49] suggested that the generation of Zn^2+^ from ZnO NPs inhibits the enzymatic action of the DapE protein involved in peptidoglycan synthesis, thus resulting in biofilm formation failure at the early stage.

Generally, biofilm formation can be classified into three main stages, namely adhesion, cell colonization, and cell maturation. The production of biofilms is initiated during the adhesion stage, where the adherent cells aggregate on the cell’s surface. Cell colonization is characterized by the formation of EPS and irreversible attachment to the surface of the host cells. Cell maturation is the stage where the biofilm reaches its maximum cell density [50,51]. In the present study, the eradication of 24 h (irreversible) and 48 h (matured) old biofilms was investigated. As shown in Figure 4b, the percentage of eradicated biofilms at 24 h ranged from 60 to 80% at all concentrations, whereas the eradication of the biofilms at 48 h ranged from 60 to 70%. This finding may be due to the production of multi-layer biofilm at the mature stage, requiring a higher concentration of ZnO NPs for its eradication. Chen et al. [52] demonstrated that *S. aureus* young biofilms (24 h) treated with antibiotics were easier to eradicate than older biofilms (72 h). This implies that matured biofilms have lower antibiotic susceptibility compared with young biofilms. Overall, both biofilms and planktonic cultures were susceptible to ZnO NPs, which makes antibacterial-based NPs a good candidate to prevent widespread biofilm formation.

### 3.5. Time–Kill Assay and Trypan Blue Exclusion Assay

**Figure 5:**
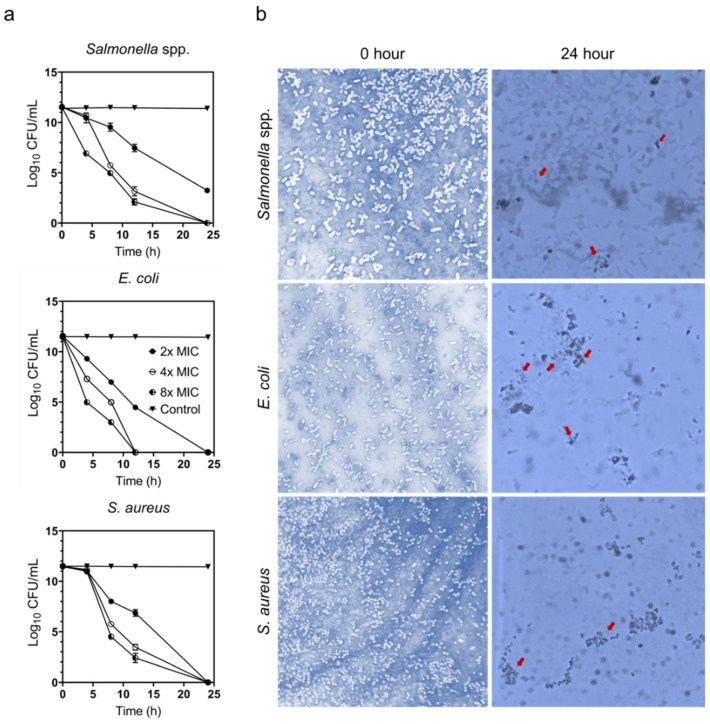
(**a**) Time–kill assay based on cell viability (log_10_ CFU/mL) on the poultry-associated foodborne pathogens, *Salmonella* spp., *E. coli*, and *S. aureus* at the concentrations of 2×, 4×, and 8× MIC at different time intervals (0, 4, 8, 12, and 24 h). Bars represent the standard deviation of the mean for three replicates. (**b**) Trypan blue dye exclusion assay of *Salmonella* spp., *E. coli*, and *S. aureus* at 0 h and 24 h treated with ZnO NPs at the concentrations of 8× MIC. Dead cells are indicated with red arrows. The 0 h selection were used as a control of live cells. The images were taken using a light microscope at 100× magnification.

The time–kill assay of ZnO NPs against the poultry-associated foodborne pathogens was assessed to determine the effect of ZnO NPs on bacterial viability and to ascertain the time required to meet the bactericidal activity threshold. The assay was carried out by suspending the bacterial cell in ZnO NPs-containing media. The suspension without the addition of ZnO NPs was used as control. Based on the results presented in Figure 5a, a rapid decrease in CFU among all bacterial cells is observed after 8 h exposure to ZnO NPs, where more than 5-fold CFU reduction is observed. Pronounced bactericidal activity is observed at higher ZnO NPs concentrations (4× and 8× MIC). The bacteria are completely killed after 24 h exposure to ZnO NPs. Interestingly, *E. coli* demonstrated a shorter time for the bactericidal effect within 12 h at 4× and 8× MIC concentrations.

In this study, it was also observed that the higher the concentration of ZnO NPs, the shorter the time required for the bactericidal effect on the tested bacteria. Xie et al. [53] demonstrated that ZnO NPs effectively killed *Campylobacter jejuni* cells in less than 3 h, even at low concentrations, indicating that the bacteria were highly susceptible to ZnO NPs. Likewise, Hoseinzadeh et al. [54] reported that the killing time for *E. coli* and *S. aureus* at 2 × MIC was 6 and 3 h. Compared to other studies, our time–kill results took a bit longer to completely kill the bacteria, possibly attributable to the variation of NPs size, bacterial species, and the method used.

A trypan blue exclusion assay was performed to verify the damage of the bacterial cell membrane. In this study, the killing effect of ZnO NPs is speculated to be associated with the damage of the cell membrane, which contributes to cell death. As a result, a trypan blue exclusion assay was performed to distinguish dead bacteria with damaged cell membranes from live bacteria with intact cells. Generally, the trypan dye penetrates the damaged cell membrane, causing the cells to appear blue under the microscope. Based on the findings obtained in Figure 5b, live bacteria displayed an intact cell membrane at 0 h, but after 24 h incubation, the bacteria appeared blue, indicating cell membrane damage. Therefore, our results suggested that ZnO NPs disrupted the membrane cell, leading to cell death.

### 3.6. Quantification of Reactive Oxygen Species (ROS) and Bacterial Cellular Leakage (Protein and Sugar)

**Figure 6:**
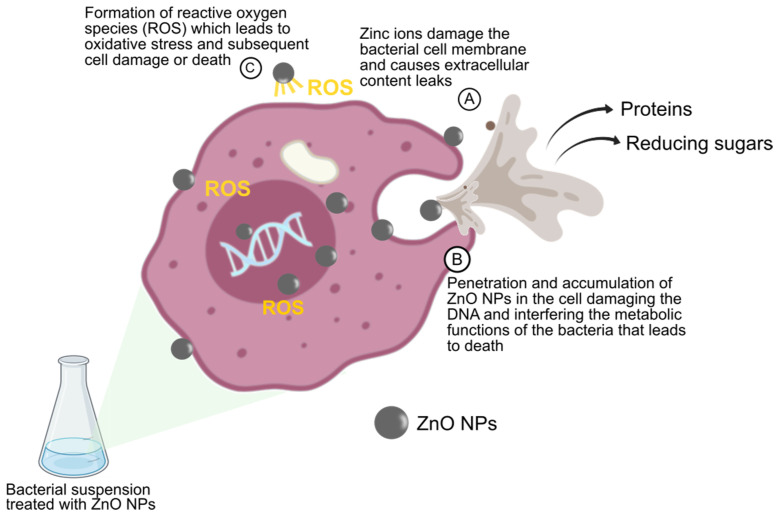
The proposed antibacterial actions of zinc oxide nanoparticles (ZnO NPs).

The production of ROS was assessed in all the bacteria by treating them with ZnO NPs. Bacterial cells produce excessive ROS, including superoxide radical, when the ambient environment is unconducive, which means more enzymes and antioxidants need to be produced to scavenge the ROS [55]. However, the accumulation of ROS results in oxidative stress-induced cell death. Various antibacterial mechanisms of NPs have been proposed [6,38] (Figure 6), with the production of ROS being the most commonly proposed antibacterial mechanism. Metal and metal oxide NPs are well documented in their ability kill microorganisms through the generation of ROS. The chain of events leading to cell death following the production of ROS from ZnO NPs includes cell membrane disruption, attenuation of DNA replication, and dysfunctional production of adenosine triphosphate (ATP), leading to impaired bacterial metabolism [56].

**Figure 7:**
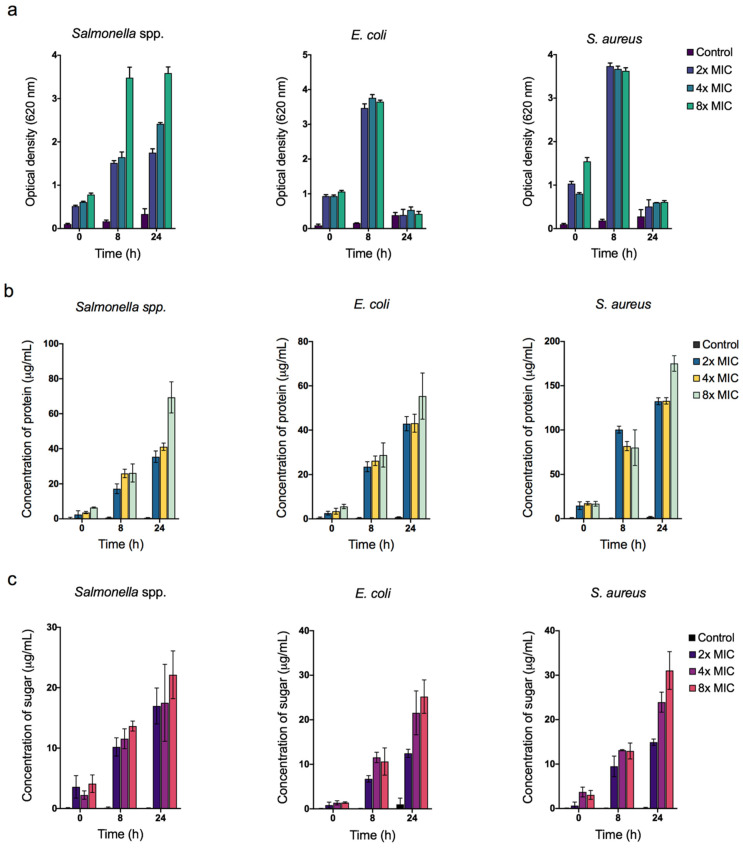
(**a**) Reactivity oxygen species (ROS) quantification. Cellular leakage analysis of (**b**) protein content and (**c**) reducing sugar from the cell suspensions of *Salmonella* spp., *E. coli*, and *S. aureus* bacteria treated with different concentrations of ZnO NPs at 0, 8, and 24 h of incubation time. Data were shown as the mean ± SD of three replicates.

In this study, the ROS concentration formed in the bacterial cell was quantified using nitro blue tetrazolium (NBT). NBT is a pale-yellow water-soluble nitro-substituted aromatic tetrazolium compound that reacts with cellular ROS in the bacterial cell to form a formazan derivative with a dark purple coloration that can be monitored spectrophotometrically [57]. Therefore, the NBT reaction can be simultaneously used for an indirect reflection of the ROS-generating activity in the bacterial cell and to quantify the amount of ROS produced. In Figure 7a, a remarkable increase in ROS level was observed in all the bacteria over the incubation time. However, the level of ROS was depleted after 24 h in *E. coli* and *S. aureus* (Figure 7a), which could be explained by the loss of ROS in the suspension. The production of ROS peaked at 8 hours, and afterward, none of the bacterial cells generated ROS. Moreover, ROS production is likely to occur during the early stage of the interaction between ZnO NPs and the bacteria. Liao et al. [58] also reported that silver NPs induced excessive ROS production in multidrug-resistant *Pseudomonas aeruginosa* in a time- and concentration-dependent manner.

Further studies revealed that cellular leakage is the possible mechanism of ZnO NPs bactericidal action. The release of cellular content such as protein and reducing sugars is considered to be a reliable indicator of cell membrane permeability and damage. As depicted in Figure 7b,c, both protein and reducing sugars content showed a similar increment pattern, where the leakage of cellular content increased with the increase in incubation time. A probable explanation may be that more cells were damaged as the incubation time increased, which lead to higher amounts of intracellular content leakage. Furthermore, the extent of cellular content leakage was more profound in ZnO NPs with the highest concentration in all the bacterial strains. This indicated that a higher concentration of ZnO NPs had the potential to kill more cells.

In conclusion, ROS exerts mechanical stress on the cell membrane, compromising the membrane integrity of bacteria, leading to cellular content leakage and cell death. A similar observation was observed by Kim et al. [59], based on the effects of silver NPs on *E. coli* and *S. aureus*. The authors reported a greater amount of protein leaked from the cell due to the generated ROS from silver NPs in bacterial cells leading to membrane permeability. Likewise, ZnO NPs have been shown to have a bactericidal effect on *Campylobacter jejuni*, a common poultry pathogen, by inducing oxidative stress through ROS in bacterial cells and disrupting the cell membrane [53]. These results were corroborated by Jiang et al. [60], who confirmed that the bactericidal activity of ZnO NPs was inhibited in the presence of radical scavenger, thus providing clear evidence that the antibacterial activity of ZnO NPs is ROS-mediated.

### 3.7. Bacterial Surface Morphology Study Using Scanning Electron Microscope (SEM)

**Figure 8:**
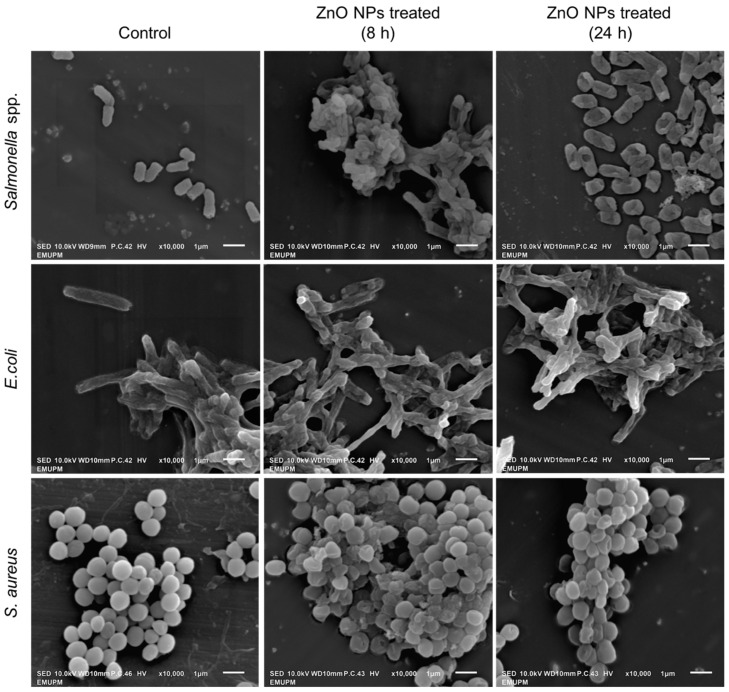
Visualization of zinc oxide nanoparticles (ZnO NPs) treated *Salmonella* spp., *E. coli*, and *S. aureus* at 8 and 24 h using a scanning electron microscope (SEM). The absence of ZnO NPs was used as control.

The damage to the bacterial cell membrane was further investigated by SEM analysis. SEM micrographs in Figure 8 illustrate several morphological changes at 8 and 24 h cell exposure time to ZnO NPs, which differed from the control group. The cell membrane of all bacterial strains appears distorted and becomes severely damaged after 24 h. The micrographs portray holes on the membrane surface of ZnO NPs-treated cells, indicating bacterial membrane deformities upon exposure to ZnO NPs. The morphological changes of all treated bacteria are similar, where the cell wall becomes crumpled, with some becoming atrophic and rupturing, leading to subsequent lethal events. Our SEM results revealed a good correlation with the trypan blue and time-killing assays (Figure 5), where no viable cells were observed after 24 h. Likewise, the formation of holes (pits) on the cell membrane caused cellular contents leakage which corroborated our protein and reducing sugars quantification results. Similarly, Wu et al. [61] reported that silver NPs showed an effective antibacterial activity against *S. aureus* and *E. coli*, as observed by the formation of pits in the cell walls. The pit formations allow the NPs to enter the periplasm and destroy the cell membrane. It also enables the NPs to penetrate the cytoplasm, causing the interaction that resulted in the leakage of cellular components [61]. Significant changes in the cell morphology of bacteria were also observed in several studies [62,63,64], congruent to our findings.

The exact mechanisms for the antibacterial action mode of ZnO NPs are still debated; however, our findings suggested that the oxidative stress caused by ZnO NPs might be one of the reasons for bactericidal activity. Another possible mechanism is the attachment of metal NPs to the cell membrane, which exerts mechanical stress on the membrane surface [65]. The main composition of the bacterial cell membrane is proteins and lipids, which are also the sites of interaction of most metal NPs. Durán et al. [66] reported that silver ions react with the thiol groups of protein, causing the inactivation of membrane-bound enzymes and proteins. Moreover, Wong and Liu [67] proposed that the small particle size also plays a significant role in the antibacterial mechanism by providing a large surface area that allows them to attach to the cell membrane and penetrate the bacteria. Moreover, the presence of metal NPs (metal ions) in the cells interferes with the respiratory chain in the bacterial mitochondria, hampering energy production, leading to cell death [67,68].

### 3.8. Overview of Risk Assessment for the Application of Zinc Oxide Nanoparticles in the Poultry Industry

In this present study, the ZnO NPs demonstrated a good in vitro antibacterial activity against Gram-positive and Gram-negative bacteria. However, the use of ZnO NPs as feed antibiotics in animals based on their destructive effects on the gut microbiota, especially on the beneficial microorganism, remains debatable. The intestine of poultry contains trillions of microorganisms that form a complex microbial community, coexisting with the host, and plays a critical role in the body’s physiological function, such as nutrition digestion, gut mucosal morphology, and immune system development [69].

Several studies reported that the supplementation of ZnO NPs on animals induced microbial alterations in the gut. For example, Reda et al. [70] reported that the dietary supplement of ZnO NPs at 0.1 g/kg on Japanese quail led to a significant increase in all cecal microbiota counts, including *Lactobacillus* and *Salmonella*, except *E. coli* and *Enterococcus* spp. However, supplementing ZnO NPs at a higher dosage of 0.4 g/kg resulted in a significant reduction in the microbiota population, indicating that the level of ZnO NPs concentration plays an important role in regulating the intestinal microbiota. Likewise, Feng et al. [14] discovered that the ileal microbiota of hens decreased with the administration of increasing ZnO NPs concentration, with a higher concentration of ZnO NPs at 100 mg/kg causing more microbiota reduction. They also found that ZnO NPs remarkably reduced the relative abundance of *Lactobacillus*. On the other hand, the reduction of *Lactobacillus* does not affect the growth performance of the hens [14]. Furthermore, bulk ZnO was traditionally used in higher doses (up to 3000 mg/kg feed) of pig nutrition to combat enterobacteria-caused post-weaning diarrhea. A recent study by Pei et al. [71] reported that the population of *E. coli* in the cecum and colon of pigs supplemented with 450 mg/kg and 3000 mg/kg of ZnO NPs and bulk ZnO was significantly decreased. Meanwhile, no decrease in the amount of *Lactobacillus* and *Bacillus bifidus* (Bifidobacteria) was reported in their research, implying that ZnO NPs reduced *E. coli* and retained the number of beneficial bacteria.

*Lactobacillus* is a predominant beneficial gut bacterial genus in the animal gut ecosystem that plays a significant role in the intestinal health and growth performance of poultry. However, it is easily impacted by various factors such as feed composition, feed additives, and antibiotic growth promoters. Nonetheless, previous studies were inconsistent, with some studies reporting no reduction of a beneficial bacteria population, notably *Lactobacillus*, vice versa. A large reduction of *Lactobacillus* in poultry’s gut was thought to be due to their dominating population, which can be easily changed. Meanwhile, the increasing number of *Lactobacillus* is probably due to its ability to reproduce rapidly in the gut ecosystem. Even though ZnO NPs caused a decrease in the gut microbiota population, supplementing animals with ZnO NPs did not cause severe growth and gut health problems. Ali et al. [72] documented that supplementing ZnO NPs at 40 mg/kg in broiler enhanced gut health by improving intestinal microarchitecture (increasing the villus height and surface area) in all segments of the small intestine and increasing the immunomodulatory effect. Subsequently, long villi and greater surface area are correlated with greater absorption of available nutrients and better gut health. Furthermore, the higher bioavailability of ZnO NPs was assumed to be responsible for the increased villus microarchitecture, which contributed to a reduction in cell turnover rate in the villi and resulting in higher villus height [72,73]. Likewise, several studies also reported that supplementation of ZnO NPs improved the intestinal health of animals [20,70,74]. Nevertheless, more investigation is required to further elucidate the antibacterial effects of ZnO NPs and ensure its potential applications as a safe regulator of gut microbiota of poultry because the result may vary depending on the segment of the intestine, dose, shape, and size of ZnO NPs.

Although ZnO NPs are widely used in many bio-applications in animals, their toxicity risk in the body remains controversial because while some studies have found that ZnO NPs have therapeutic benefits, others reported them to be toxic to living organisms. Nonetheless, investigations have shown that dose [75], size, shape [76], and functionalized groups [77] of ZnO NPs are the key factors contributing to the toxic effects. In addition, chemically manufactured ZnO NPs were demonstrated less biocompatible due to the use of toxic chemicals for stabilizing agents that may reside in the final product of NPs, becoming one of the factors that leads to innate toxicity of NPs. ZnO NPs in this work were produced using the biological route without the inclusion of toxic chemicals, which may reduce the toxicity effects of NPs. Furthermore, administering higher concentrations or doses of ZnO NPs to the animals may also result in toxicity. For example, Wang et al. [78] found that supplementing mice with high doses of ZnO NPs at 5000 mg/kg caused toxicity by reducing body weight and increasing the relative weight of organs. Therefore, the use of ZnO NPs in animal feed should be limited to a specific minimum concentration to avoid this hazardous effect, which necessitates additional research. In addition, it is critical to assess ZnO NPs cytotoxicity in both cancer and normal cell lines because much of the current literature documented cytotoxicity effects on cancer cell lines [79] and claims that ZnO NPs are toxic to all cells might be deceptive, as it may not be hazardous to healthy cells.

Several studies reported that metal and metal oxide NPs have excellent antibacterial activity against multidrug-resistant bacteria [1,58,63,64]. However, one of the biggest issues with using NPs is the possibility for bacteria to develop metal-tolerance mechanisms, particularly in the gut ecosystem of animals and in the environment. To date, there has been a lack of research carried out on the emergence of metal-resistant bacteria. Nonetheless, some research has discovered that bacteria’s tolerance to NPs may be due to electrostatic repulsion, biofilm adaptation to NPs, and ion efflux pumps in the bacterial system [80]. Furthermore, earlier research has only identified mechanisms of tolerance to NPs in in vitro exposure studies, although such mechanisms will differ in vivo, particularly in the intestinal environment of animals [81]. Therefore, additional research is required to emphasize the toxicology and possible development of metal-resistant bacteria, concerning the type of metal NPs, level of concentration, and the method used.

The widespread usage of ZnO NPs in various industries has raised concerns regarding their environmental impact. Excessive usage of ZnO NPs would inevitably release many NPs into the environment, resulting in heavy metal contamination. In the animal feed industry, ZnO NPs are introduced into poultry feed in a small amount due to their small particle size, which improves bioavailability and Zn absorption in the body. In this sense, no or low quantities of ZnO NPs will be discharged into the environment, reducing heavy metal contamination. However, a continuous and dynamic risk assessment is still required. To summarize, the safety and risk of the application of ZnO NPs in the animal industry, particularly poultry, should be assessed carefully before the direct implementation of ZnO NPs for any application.

## 4. Conclusions

In the present study, we reported the antibacterial activity of biologically synthesized ZnO NPs against poultry-associated foodborne pathogens, *Salmonella* spp., *E. coli*, and *S. aureus*. The ZnO NPs were synthesized by using *L. plantarum* TA4, followed by the analysis of the physicochemical characteristics. The biosynthesized ZnO NPs had spherical shapes with an average particle size of 29.7 nm. The Zn^2+^ dissolution study revealed that ZnO NPs dissolved more ions than their bulkier counterparts, implying that the release of high Zn^2^ was responsible for the antibacterial activity. Based on the agar well diffusion assay, MIC and MBC, time–kill assay, and antibiofilm activity tests, the ZnO NPs exhibited effective antibacterial actions against poultry-associated foodborne pathogens, with *S. aureus* as the most susceptible to ZnO NPs. The ROS formation, cellular leakage, and SEM study revealed that the underlying antibacterial mechanisms of ZnO NPs include the generation of ROS, oxidative stress on the bacterial cell membrane leading to membrane damage, and cellular material leakage, which ultimately leads to cell death. In conclusion, the results of this in vitro antibacterial study demonstrated that biosynthesized ZnO NPs have great potential to be used as an alternative antibacterial agent (nanobiotic) in poultry production to control the gut burden of poultry-associated foodborne pathogens. Finally, further studies are needed to elucidate the in vivo antibacterial efficacy in poultry production.

## Figures and Tables

**Table 1:** Antibacterial activity of bulk ZnO and zinc oxide nanoparticles (ZnO NPs) against *Salmonella* spp., *E. coli*, and *S. aureus* at various concentrations. The antibacterial activity was measured by the inhibition zone in millimeters (mm).

Concentration (µg/mL)	Zone of Inhibition (mm)					
	*Salmonella* spp.		*E. coli*		*S. aureus*	
	Bulk ZnO	ZnO NPs	Bulk ZnO	ZnO NPs	Bulk ZnO	ZnO NPs
1000	ND	8.00 ± 0.00 ^d^	ND	8.00 ± 0.00 ^c^	7.33 ± 0.58 ^c,y^	11.33 ± 1.15 ^c,x^
2000	ND	9.33 ± 0.58 ^c^	ND	9.00 ± 1.00 ^b^	7.67 ± 0.58 ^c,y^	12.00 ± 1.00 ^c,x^
3000	ND	10.67 ± 0.58 ^b^	ND	10.33 ± 0.58 ^a,b^	8.67 ± 0.58 ^b,y^	15.00 ± 1.00 ^b,x^
4000	ND	12.00 ± 0.00 ^a^	7.00 ± 1.00 ^a,y^	11.00 ± 1.00 ^a,x^	9.67 ± 0.58 ^a,y^	16.00 ± 1.00 ^b,x^
5000	ND	12.33 ± 1.53 ^a^	8.00 ± 0.00 ^a,y^	12.00 ± 1.00 ^a,x^	10.00 ± 1.00 ^a,y^	19.67 ± 0.58 ^a,x^

^a–d^ Mean values with different superscripts in the same column corresponding to each type of bacteria are considered statistically different (*p* < 0.05). ^x,y^ Mean value with different superscripts in the same row corresponding to different concentrations of ZnO NPs are considered statistically different (*p* < 0.05). Values are mean ± SD error of three replicates. ND = No diameter for inhibition zone.

**Table 2:** Minimum inhibitory concentration (MIC) and minimum bactericidal concentration (MBC) values of zinc oxide nanoparticles (ZnO NPs) and their bulk forms against *Salmonella* spp., *E. coli*, and *S. aureus*.

Bacteria	Bulk ZnO		ZnO NPs	
	MIC (µg/mL)	MBC (µg/mL)	MIC (µg/mL)	MBC (µg/mL)
*Salmonella* spp.	200	800	80	160
*E. coli*	200	1000	60	140
*S. aureus*	100	800	30	100

## Data Availability

The data presented in this study are available on request from the corresponding author.
